# The effects of shared decision-making compared to usual care for prostate cancer screening decisions: a systematic review and meta-analysis

**DOI:** 10.1186/s12885-018-4794-7

**Published:** 2018-10-22

**Authors:** Nahara Anani Martínez-González, Stefan Neuner-Jehle, Andreas Plate, Thomas Rosemann, Oliver Senn

**Affiliations:** Institute of Primary Care, University of Zurich and University Hospital of Zurich, Pestalozzistrasse 24, CH-8091 Zurich, Switzerland

**Keywords:** Systematic review, Meta-analysis, Shared decision-making, Prostate Cancer, Screening, Randomised controlled trials

## Abstract

**Background:**

Shared decision-making (SDM) is recommended for men facing prostate cancer (PC) screening decisions. We synthesize the evidence on the comparative effectiveness of SDM with usual care.

**Methods:**

We searched academic and grey literature databases, and other sources for primary randomised controlled trials (RCTs) published in English comparing SDM to usual care and conducted in primary and specialised care. We assessed the individual study risk of bias, and calculated the study-specific and pooled relative risks (RR) or standardised mean differences (SMD) [with 95% confidence intervals (CI)] to perform random-effects meta-analyses for SDM-related and patient outcomes.

**Results:**

Four RCTs comparing SDM to usual care, involving 1760 men, were included. SDM improved knowledge (SMD 0.23, 95%CI 0.02 to 0.43; 2 RCTs), but was not different to usual care in reducing either patient participation in prostate-specific antigen (PSA) testing (RR 1.03, 95%CI 0.90 to 1.19; 2 RCTs) or decisional conflict (SMD -0.04, 95%CI -0.23 to 0.15; SMD -0.05, 95%CI -0.24 to 0.14; 2 RCTs). Individual trial estimates (46.7%) also suggest that SDM may reduce or neutralise physicians’ tendency for PSA screening, and may improve the accuracy of patients’ perception of lifetime-risks and men’s views towards screening. There was no evidence on the effects of SDM on health outcomes. The studies represent various interventions and outcomes and are prone to risk of bias.

**Conclusions:**

There is currently insufficient evidence to support a clear association of SDM on patient- and SDM-related outcomes for decisions about PSA testing. Further research needs to assess the clinical effectiveness of SDM using well-defined SDM interventions and outcomes. It should address the absence of evidence, particularly on health outcomes.

**Electronic supplementary material:**

The online version of this article (10.1186/s12885-018-4794-7) contains supplementary material, which is available to authorized users.

## Background

Prostate cancer (PC) is the second most commonly diagnosed non-skin malignancy and the fifth leading cancer-related cause of death for men worldwide [1, 2]. PC incidence varies mainly by age, race/ethnicity and family history [2, 3]. It continues to rise, mostly in Western developed countries [1, 4] and is expected to increase to 1.7 million cases and 499,000 new deaths by 2030 globally [2].

Screening for PC aims to diagnose the disease at an early stage when the chances of successful treatment are higher, thus increasing the possibility of cure. Indeed the widespread use of screening tests, especially prostate-specific antigen (PSA) in the general population, has improved early PC detection, thereby increasing the incidence of diagnosed PC. The balance between the benefits and harms of screening remains controversial however [5]. PC testing has led to false-positive results, complications and over diagnosis (risk estimates: up to 67%) that may lead to further unnecessary investigations and overtreatment [6]. Data about the reduction in mortality due to PC screening have also shown conflicting results. Population-based trials have shown no significant differences in PC mortality after 13, 15 or 20 years [7–9], while others showed reduced risks of metastases and PC-specific mortality after 11 and 13 years [10–12]. In addition, treatment for screened-detected PC can lead to potential adverse outcomes (e.g. urinary and erectile dysfunction, loss of fertility, chemotherapy and/or hormone therapy side effects), distress, impaired quality of life, and increased healthcare costs [10, 13, 14]. Guidelines for PC screening consequently vary worldwide. Recommendations for PC testing by some health authorities are becoming more consistent by setting constraints and only screening well-informed men [15–19]. Others have developed specific population-based screening programs [20]. Some others are against population-based screening, but still provide testing on demand [21–23]. In addition, the screening practices for men at risk of PC and the age at which screening should be started for example, are still being debated [24]. These factors, together with the fast-growing availability of cancer testing and treatment technology, make the process of medical decision-making even more challenging for both patients and healthcare professionals (HCP), leading to value-laden decisions that are preference-sensitive.

Shared decision-making (SDM) is viewed as the best practice model for physician-patient-communication regarding preference-sensitive medical decisions [25]. Experts, major task force associations, policy makers and clinical guidelines strongly advocate SDM discussions as a critical step preceding medical decisions for PC screening [15, 17, 19, 22–24, 26]. SDM may have the potential of reducing the overuse of options with unclear benefits while enhancing the use of beneficial options and reducing variations in practice [27, 28]. To-date, however, there is no single definition of SDM and no consensus on how to integrate SDM in practice resulting in varying levels of SDM implementation. A systematic review showed that key criteria recommended for SDM [29] supporting the principle of deliberation [30] was fulfilled by only 34.8% of the studies evaluating SDM for decisions about PC screening [31].

Increasing research has focused on the development of decision aids (DAs) as a way to promote informed medical decisions and to improve patient outcomes [32]. Evidence on the effects of SDM is not restricted to DAs however. In addition, provision of DAs does not ensure patient participation in decision-making nor does it warrant an SDM approach to medical decisions. The role of SDM in improving patient outcomes as compared to usual care remains unclear. We sought to evaluate the evidence on the effectiveness of SDM as compared to current clinical practice for patient- and SDM-related outcomes. We focused on the population of men facing preference-sensitive decisions for PC screening.

## Methods

Our study followed a protocol based on the recommendations for systematic reviews of healthcare interventions [33, 34] and the PRISMA guidelines (Additional file 1) [35]. The methods to identify and select studies are described in detail elsewhere [31].

### Search strategy

Using terminology compatible with SDM and prostate cancer, we searched for RCTs using MEDLINE Ovid, EMBASE (Elsevier), CINHAL (EBSCOHost), The Cochrane Library (Wiley), PsychINFO (EBSCOHost) and Scopus from the period of database inception up to March 2015 (see Additional file 2). We searched for grey literature using clinical trial registers (clinicaltrials.gov and ISRCTN), the WHO search portal (http://www.who.int/ictrp/search/en/) and the Ottawa Hospital Research Institute website (http://www.ohri.ca//) by accessing the records between February and August 2016, and searching for the trials registration number in Medline and PubMed. We also searched Google Scholar and the system for Information on Grey Literature in Europe (http://opengrey.eu/). Additionally, we screened the reference lists of included studies, relevant systematic reviews and clinical guidelines. We also contacted (June 2015 to January 2017) the authors of abstracts for which full-text publications could not be located.

### Eligibility criteria

We included peer-reviewed randomised controlled trials (RCTs) published in English, from any country, investigating SDM for PC screening in primary or specialised care (general practice, community clinics, ambulatory care, hospital and private care services), which: 1) compared SDM to usual care regardless of the intervention target (patients and/or HCP, surrogates or family members), and 2) reported outcome data in quantitative format. We further limited study eligibility to RCTs that fulfilled the criteria for SDM based on the most prominent SDM framework [29, 36] considering bi-directional deliberation as central and mandatory in SDM [37]. As detailed elsewhere, this process led to a set of studies of higher SDM quality [31].

Outcomes’ relevance is generally directed by the personalisation of the SDM process, patients’ health status and the available choices, resulting in a non-standardised set of outcomes to evaluate the success of SDM. We focused on the outcomes of knowledge, screening participation, risk perception, patient satisfaction, decisional conflict, decisional regret, quality of life, symptoms and mortality.

### Selection of studies and data extraction

Two authors independently screened the titles and abstracts of all citations, and examined the full-text of eligible publications. Data extractions were carried out by one author, and independently verified by a second author. Discrepancies in study selection and data extractions were moderated by a third author. Data were extracted using standardised data collection forms developed a-priori. For each study, one author extracted data elements including the bibliographic details, design, country and setting, characteristics of study populations and interventions, and outcome data. Studies reported across more than one publication were treated as one unit. We used the classification system recommended by Shay and Lafata [38] to group outcomes into Affective-cognitive, Behavioural and Health outcomes.

### Study quality

Two authors independently assessed in duplicate the quality features of the included studies without the calculation of a composite score following available recommendations [34, 39]. Differences were resolved by discussion. We rated the adequacy of the studies’ core items for internal validity (risk of bias) including generation of random sequence, concealment of allocation at randomisation, blinding (patients, healthcare providers and outcome assessors), intention-to-treat (ITT) and similarity between groups at baseline. We considered bias due to attrition of at least 20% to be of significant concern; adequate ITT when authors analysed participants based on their original group allocation; and adequate follow-up if all participants were followed-up for the same length of time. We also assessed whether studies defined primary and secondary outcomes, inclusion and exclusion criteria, ‘a-priori’ sample size and the type of funding sources.

### Statistical analysis

Where data were sufficiently reported, for each study outcome we calculated the unadjusted risk ratios (RR) or the standard mean differences (SMD) and the 95% confidence intervals (CI) with RevMan, version 5.3.5 (http://community.cochrane.org/). We pooled data in meta-analyses when at least two RCTs reported appropriate data. Missing standard deviations (SD) were estimated using established techniques [34]. We report the summary statistics with their 95% CI, and regard *p* < 0.05 as statistically significant. We quantified heterogeneity using the I^2^ statistic, for which values of 25%, 50%, and 75% represent low, moderate, and high levels of heterogeneity [40]. Data not combined in meta-analyses were analysed based on the individual trial estimates. We anticipated heterogeneity due to diversity in populations and interventions and thus performed all analyses under random-effects [34]. We tabulated the effect sizes and synthesised all results narratively.

## Results

### Identification of studies

**Fig. 1:**
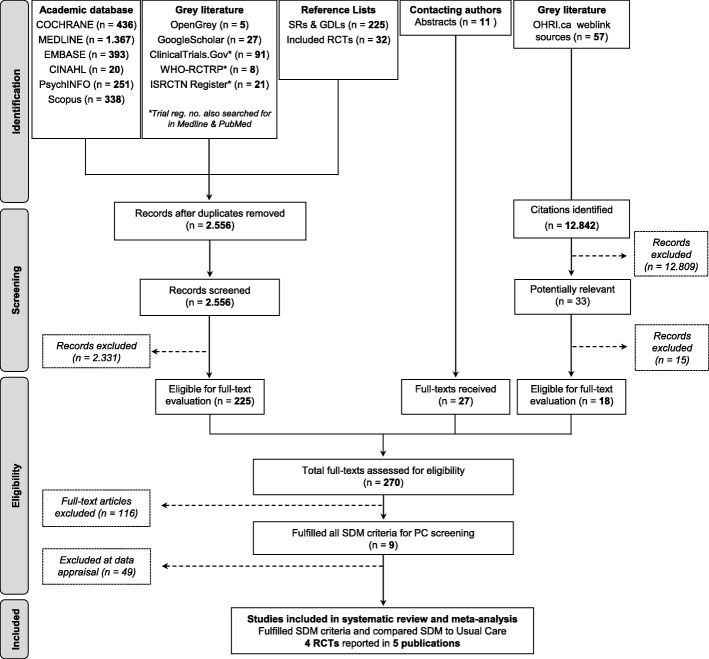
Identification and selection of studies

Of 270 full-text articles examined in detail, nine fulfilled all SDM criteria. Five of these investigated the comparative effectiveness of SDM with usual care [41–45]. Figure 1 shows the flow of study identification and selection.

### Study and population characteristics

The summary characteristics of the included studies are reported in Table 1. The studies, published between 2003 and 2013, were conducted in the USA (*n* = 3) and Australia (*n* = 1). Three parallel-RCTs (75%) comprised 1048 patients individually randomised to intervention groups while one cluster-RCT randomised 712 patients with 120 physicians and 55 waiting areas. Participants were recruited from general or internal medicine primary care clinics including urban/suburban, academic, medical group practices or university-hospital affiliated clinics. All studies defined PC screening as testing with PSA. The men’s age range was 54–63 years with a mean age of 59.5 (SD7.5; 3 RCTs) and the target-age for study inclusion was 40–74 years. Patient demographics varied widely across studies. Where reported, 76.6% (range: 56.6–90.8%) of men were White, 76.9% (range: 71.5–80.0%) were married, 76.45% (range: 30.4–89.5%) had at least high school education, and 46.9% (range: 42.5–57.5%) were in full- or part-time employment. At least 56.7% of all participating men were screened with PSA before study enrolment, and 12.4% of men in three RCTs reported a family history of PC. Between 13 and 120 family or internal medicine (i.e. faculty, resident and academic) physicians participated in the studies and had 2–40 years of experience in two RCTs.

**Table 1:** Summary characteristics of studies included in review

First author, publication year, country, design & period of conduct	Healthcare context, setting and facilities, n	Target population	Total randomised, N	Intervention & randomised patients, N	Comparator(s) & randomised patients, N	Age: mean (SD) & target (range), years	Race or ethnicity, %			Ever screened, %	Family history of PC, %	Married, %	Education: >HS, %	Employment in full- or part-time, %	Participating HCP & specialty, n
							White	Black	Hispanic						
Wilkes, 2013 [41] USA RCT, cluster May 2007 to Dec 2008	General medicine Primary care networks academic-medical-centre affiliated, 2 Staff model health maintenance organisations, 2 Medical group practice network, 1	Men with no serious comorbidity (including any known cancer) and English speakers. Physicians consented to participate in educational activities and to help recruit patients	55 waiting areas, 712 patients, 120 physicians	1) MD-Ed + A: interactive web-based physician educational program (30 min) with information about PC and screening + web-based patient activation (30 min) + access to CDC brochure; *N* = 19 waiting areas, 113 patients, 36 physicians 2) MD-Ed: interactive web-based physician educational program (30 min) with information about PC and screening + access to CDC brochure; *N* = 19 waiting areas, 246 patients, 41 physicians	CDC educational brochures; *N* = 17 waiting areas, 353 patients, 43 physicians	63.2 (7.0) (55–65)	74.5	7.1	7.1	82.7	18.4	80.0	89.5	42.5	Internal and family medicine physicians with ≥4–40 years’ experience since clinical training completed, 120
Landrey, 2013 [42] USA RCT, parallel Oct 2009 to Aug 2010	General medicine General internal medicine practices University-Hospital affiliated, 2	Men scheduled to have an annual health maintenance exam between October 2009 and August 2010	303	Flyer about PC and PSA screening with patient encouragement to talk with providers; *N* = 145	No flyer; *N* = 158	62.0 (nr) (50–74)	56.5	5.3	2.1	0.0	0.0	75.6	nr	nr	Internal medicine physicians, 44
Krist, 2007 [43, 44] (Woolf, 2005) USA RCT, parallel Jun 2002 to Jun 2004	General medicine Suburban family practice centre, 1	Men with a scheduled health maintenance examination	497	1) Web-based informational DA about PC and PSA screening; *N* = 226 2) Pamphlet, paper version of web-based DA (with same information); *N* = 196	No pre-visit educational material and no DA during discussions with physicians; *N* = 75	56.6 (4.0) (50–70)	90.8	2.6	0.0	68.5	0.0	nr	84.1	nr	Family physicians, 29: 13 faculty, 8 second-year residents, 8 third-year residents
Gatellari, 2003 [45] AUS RCT, parallel Period, nr	General medicine Urban general practices, 13	Men sufficiently fluent in English, not diagnosed with PC, from 13 GPs in urban Sydney	248	32-page (3085-word) evidence-based informational booklet about PC and PSA screening in quantitative data form with maximised readability with Flesch–Kincaid grade level = 7.3; *N* = 126	968-word pamphlet by the Australian government with information to advise men of the agreed policy about PSA screening, in non-numerical data form with Flesch–Kincaid grade level = 11.2; *N* = 122	54 (8.6) (40–70)	nr	nr	nr	36.3	nr	71.5	30.4	57.5	Family physicians, 13

*PC* Prostate Cancer, *GP* General Practitioners, *PSA* Prostate Specific Antigen, *CDC* Centers for Disease Control and Prevention, *DA* Decision Aid, *nr* not reported, *MD-Ed + A* Physician Education and patient Activation, *MD-Ed* Physician Education, *HS* High School, *HCP* Healthcare Professionals

### Intervention characteristics

All RCTs and study interventions aimed at and contained elements to facilitate or foster SDM between patients and physicians (Table 1). All RCTs also fulfilled the three key SDM features [29, 36] and bi-directional deliberation [37] as previously illustrated [31]. The interventions were delivered before decision-making, either within the time of scheduled visits [43, 44] or before consultations [41, 42, 45] as an attempt to empower patients and to encourage participation in decision-making. Men were recommended to review the material before consultation [41, 43, 44], to discuss their concerns with their doctors [45] or were specifically activated [41]. Three RCTs [42–45] used patient-directed interventions, and one RCT [41] used patient-physician targeted interventions. Only one RCT used a multifaceted strategy [41]. The interventions were self-administered, and delivered on-site [41, 45] or at home [42–44], in a web-based or (printed) paper-based material or both. Only two RCTs considered healthcare literacy for the development or pilot testing of interventions [42, 45]. Two RCTs evaluated two different SDM interventions and usual care [41, 43, 44]. Their content included mostly educational information about PC and screening for physicians and patients as well as the links to informational material from established organisations (e.g. the Centers for Disease Control and Prevention). The content of usual care was incompletely described in most studies.

### Risk of bias in the methods of included studies

No trial fulfilled all the core criteria of internal validity, based on current standards [34] (Table 2). The trials were at risk of selection bias as only two (50%) had adequate random sequence generation and allocation concealment. Studies were also vulnerable to performance and detection biases since blinding of HCP and participants, and outcome assessors were adequate in only two (50%) and one (25%) RCTs respectively. Two RCTs (50%) had at least 20% attrition for some outcomes and only two (50%) reported the use of ITT techniques to deal with missing data. One RCT (25%) was potentially at risk of chance bias although it purposely had more physicians knowing the group in which patients were allocated (ratio of 1:3:3 between groups) in order to free other potential biases. Men in one RCT were followed-up in 6–16 weeks. Reporting of participants’ inclusion and exclusion criteria and calculation of sample size and power were adequate in two (50%) and one (25%) RCTs respectively. All RCTs measured the interventions’ success by definition of primary outcome(s), but data were under-reported for some outcomes in three RCTs. Non-profit institutions funded all RCTs.

**Table 2:** Methodological features of included studies

First author & publication year	Country, design & funding	Outcome definition	Inclusion (1) & exclusion (2) criteria	Study size	Sample size calculation and power	Comparable at baseline	Adequate random sequence generation	Adequate allocation concealment	Adequate blinding	Adequate follow-up	Attrition, % (range)	ITT data
Wilkes, 2013 [41]	USA, cluster RCT, non-profit	Primary & Secondary	1	*N* ≥ 200	yes	yes	unclear	yes	HCP: yes Participants: yes OA: unclear	no^a^	≤20% (0–20%)	yes^b^
Landrey, 2013 [42]	USA, parallel RCT, non-profit	Primary & Secondary	1, 2	N ≥ 200	nr	yes	unclear	nr	HCP: nr Participants: nr OA: yes	yes	≥20% (6.60–51.16%)	no
Krist, 2007 [43, 44](Woolf, 2005)	USA, parallel RCT, non-profit	Primary & Secondary	1, 2	N ≥ 200	nr	Partial^c^	yes	yes	HCP: no Participants: nr OA: unclear	yes	< 20% (0–13.29%)^d^	yes^b^
Gatellari, 2003 [45]	Australia, parallel RCT, non-profit	Primary	1	N ≥ 200	ir^e^	yes	yes	yes	HCP: yes Participants: yes OA: unclear	yes	≥20% (13.71–27.82%)	no

*ir* incomplete reporting, *nr* not reported, *HCP* Healthcare Professionals, *OA* Outcome Assessors, *ITT* Intention To Treat

^a^Follow-up was driven by the timing of the standardised patient visit and varied from 6 to 16 weeks depending on the study arm

^b^Wilkes 2013: ITT for physician-reported screening behaviour and role in decision-making, doctors’ recommendations towards screening, physician-reported outcomes. Krist 2007: ITT for decisional conflict, PSA tests ordered by physicians or self-reported by patients, but unclear for other outcomes

^c^More physicians from the website and brochure groups reported to know the group patients were in, with a ratio of 1:3:3 between groups. This was intentionally done to be free of other potential biases

^d^Unclear for two outcomes

^e^Reported on power only

### Effectiveness of SDM interventions on outcomes

Five of the nine outcomes of interest were reported in quantitative format (Table 3). Data were sparsely reported across study outcomes limiting the ability to conduct meta-analyses for most cases. Table 4 shows the individual effect estimates from trial data not pooled in meta-analyses.

**Table 3:**
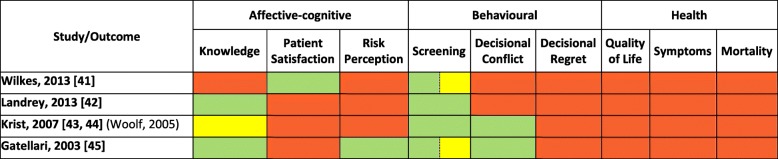
Outcomes reported in the included studies

Green = quantitative data; Yellow = qualitative data; Red = no outcome data

**Table 4:** Individual trial estimates not combined in meta-analyses

First author & publication year	Outcome	Measurement point	Intervention			Control			Effect estimate
			SDM	mean (SD)	Total (N)	Usual Care	mean (SD)	Total (N)	SMD (95 % CI)
BINARY DATA									
*Patient-reported ordering of screening*									
Krist, 2007 [43, 44] (Woolf, 2005)	patient-reported PSA tests ordered (patients' exit questionnaires)	immediately after consultation	1) web-based DA	176	226	no pre-visit educational material and no DA during discussions with physicians	60	75	0.97 (0.85 to 1.11)
			2) paper version of DA in 1)	151	196		60	75	0.96 (0.84 to 1.10)
*Actual ordering of screening*									
Landrey, 2013 [42]	PSA tests order by clinicians (chart-documented)	following doctor's appointment	flyer	85	136	no flyer	86	147	1.07 (0.88 to 1.29)
Krist, 2007 [43, 44] (Woolf, 2005)	physician-reported PSA tests ordered (chart-documented)	immediately after consultation	1) web-based DA	176	205	no pre-visit educational material and no DA during discussions with physicians	66	70	0.91 (0.84 to 0.99)
			2) paper version of DA in 1)	155	182		66	70	0.90 (0.83 to 0.98)
*Physicians' recommendations: towards screening*									
Wilkes, 2013 [41]	doctor's recommendations towards PSA screening: unannounced standardised patients (physicians’ questionnaires)	after clinic visit^b^	1) MD-Ed + A	16	36	CDC educational brochures on PC	34	43	0.56 (0.38 to 0.84)
			2) MD-Ed	24	41		34	43	0.74 (0.55 to 1.00)
*Physicians' recommendations: neither nor against screening*									
Wilkes, 2013 [41]	doctors neither suggested nor recommended for or against PSA test: unannounced standardised patients (physicians’ questionnaires)	after clinic visit^b^	1) MD-Ed + A	18	36	CDC educational brochures on PC	6	43	3.58 (1.59 to 8.06)
			2) MD-Ed	14	41		6	43	2.45 (1.04 to 5.76)
*Patient-estimates of lifetime risks*									
Gatellari, 2003 [45]	how likely men were to give a correct estimate (within 2%) of the lifetime risk of dying from PC (correct answers over incorrect answers)	unclear (questionnaires mailed 3 days post-consultations)	32-page (3085-word) evidence-based booklet	55	104	968-word pamphlet by the Australian government	3	75	13.22 (4.30 to 40.66)
	how likely men were to give a correct estimate (within 10%) of the lifetime risk of developing PC (correct answers over incorrect answers)			59	104		18	108	3.40 (2.16 to 5.36)
CONTINUOUS DATA									
*Satisfaction with the visit*									
Wilkes, 2013 [41]	patient-reported satisfaction with the visit: planned visits (sum of 5 satisfaction items: 5 = least satisfied, 20 = most satisfied)	after clinic visit^b^	MD-Ed + A	18 (3.00)	102	CDC educational brochures on PC	18 (3.00)	291	0.00 (-0.23 to 0.23)
	patient-reported satisfaction with the visit: clinic visits by patients (sum of 5 satisfaction items: 5 = least satisfied, 20 = most satisfied)		MD-Ed	18 (2.00)	188		18 (3.00)	291	0.00 (-0.18 to 0.18)
*Men's views towards screening*									
Gatellari, 2003 [45]	men’s views weighted towards or against reasons for having PSA testing (Scoring -5 to 5. Positive: weighting for; Higher: stronger weighting for; Negative: weighting against; Lower: stronger weighting against)^b^	unclear (questionnaires mailed 3 days post-consultations)	32-page (3085-word) evidence-based booklet	1.70 (1.58)	106	968-word pamphlet by the Australian government	1.4 (1.59)	108	0.19 (-0.08 to 0.46)
*Decisional conflict*									
Gatellari, 2003 [45]	decisional conflict (9-item factors contributing to uncertainty scale; higher scores = greater decisional conflict)	unclear (questionnaires mailed 3 days post-consultations)	32-page (3085-word) evidence-based booklet	21.60 (4.73)	106	968-word pamphlet by the Australian government	24.3 (4.77)	108	-0.57 (-0.84 to -0.29)

*PC* Prostate Cancer, *SDM* Shared Decision-Making, *MD-Ed + A* Physician Education and patient Activation, *MD-Ed* Physician Education, *DA* Decision Aid, *CDC* Centers for Disease Control and Prevention, *PSA* Prostate Specific Antigen, *n* number of patients with events or number of events, *N* total number of patients per group, *RR* Relative Risk, *SD* Standard Deviation, *SMD* Standard Mean Difference, *CI* Confidence Intervals

^a^Questionnaire adapted from an attitudinal measure of the mammography screening instrument

^b^Men followed-up in 6-16 weeks depending on the timing of the standardised visit: about 6 weeks after the intake survey for control physicians, 6-10 weeks for MD-Ed physicians, and 6-16 weeks for MD-Ed+A physicians

#### Affective-Cognitive outcomes

##### Knowledge

**Fig. 2:**
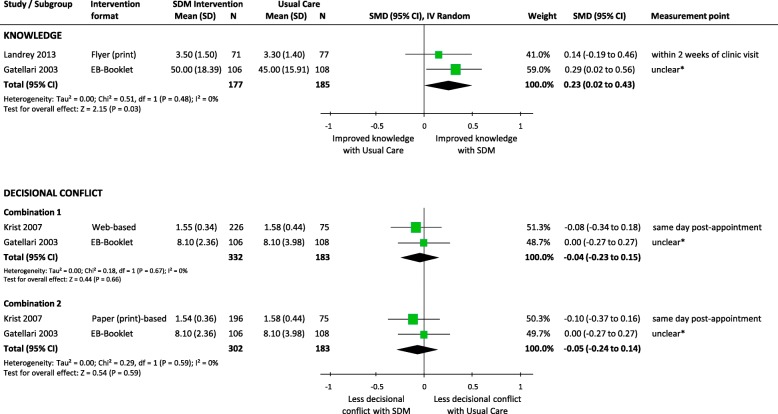
Comparison of knowledge and decisional conflict between SDM and Usual Care for men facing decisions for prostate cancer screening. SDM, Shared Decision Making; SD, standard deviation; *N*, total number of patients in the analysis; SMD, standard mean differences; CI, confidence interval; df, degrees of freedom; I^2^, heterogeneity between trials; EB, Evidence-Based. * Questionnaires were mailed three days post-consultations

Meta-analysis of two RCTs demonstrated a small but significant effect of SDM, compared to usual care, on improving knowledge of PC (e.g. natural history and risk factors) and screening (SMD 0.23, 95%CI 0.02 to 0.43, *p* = 0.03; I^2^ = 0%) (Fig. 2). There was no significant heterogeneity between trials (I^2^ = 0%, *p* = 0.48).

##### Patient satisfaction

Individual trial effect estimates showed no significant differences between usual care and SDM (with or without web-based patient activation) intervention groups with respect to the level of satisfaction with medical visits [41].

##### Risk perception

Individual trial effect estimates showed that significantly more men in the SDM group were likely to give a correct estimate of the lifetime risk of developing PC (incidence - correct within 10%: RR 3.40, 95%CI 2.16 to 5.36, *p* = 0.00001) and dying from PC (mortality - correct within 2%: RR 13.22, 95%CI 4.30 to 40.66, p = 0.00001) [45].

#### Behavioural outcomes

##### PSA testing for prostate cancer

**Fig. 3:**
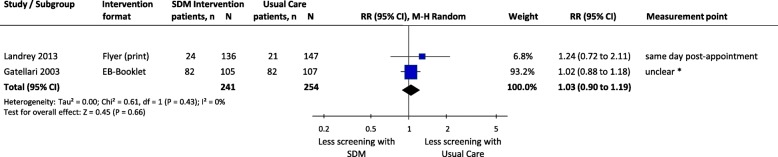
Comparison of men’s intention/preference/interest for screening between SDM and Usual Care for men facing decisions for prostate cancer screening. SDM, Shared Decision Making; SD, standard deviation; *N*, total number of patients in the analysis; RR, relative risks; CI, confidence interval; df, degrees of freedom; I^2^, heterogeneity between trials; EB, Evidence-Based. * Questionnaires were mailed 3 days post-consultations

Meta-analysis of two RCTs demonstrated no significant differences between SDM and usual care in the number of men who preferred or were interested in undergoing individualised PSA testing, with no significant heterogeneity between trials (RR 1.03, 95%CI 0.90 to 1.19, *p* = 0.66; I^2^ = 0%) (Fig. 3).

Individual effect estimates of two trials showed similar results (Table 4). In one RCT, the number of chart-documented patients for whom PSA tests were ordered by clinicians was not significantly different between groups [42]. Another RCT showed no significant differences between SDM intervention groups and usual care with respect to the number of patients who ordered PSA tests [43]. In the same RCT however, physicians reported significantly less men with ordered PSA tests in the SDM intervention groups (Web-based DA vs. Usual Care: RR 0.91, 95%CI 0.84 to 0.99, *p* = 0.02; Paper-based vs. Usual Care: RR 0.90, 95%CI 0.83 to 0.98, p = 0.02).

In one cluster-RCT, the individual effect estimates showed significantly less physicians in the SDM group with patient activation making recommendations towards PSA testing (RR 0.56, 95%CI 0.38 to 0.84, *p* = 0.004) [41]. Although the trial showed a similar pattern for the SDM group without patient activation, the effect was only marginally significant (RR 0.74, 95%CI 0.55 to 1.00, *p* = 0.05). Similarly, more physicians in the SDM group with patient activation were more neutral in recommending PSA testing (RR 3.58, 95%CI 1.59 to 8.06, *p* = 0.002) than physicians in the SDM group with no patient activation (RR 2.45, 95%CI 1.04 to 5.76, *p* = 0.04), although both were statistically significant. In another RCT, the effect estimates suggested a small, but not significant effect that men in the SDM group tend to weigh their views towards reasons in favour of undergoing PSA testing [45].

##### Decisional conflict

Meta-analysis of two RCTs demonstrated no significant differences between SDM and usual care in the level of conflict or uncertainty in making medical decisions (Combination 1, SMD -0.04, 95%CI -0.23 to 0.15, *p* = 0.66; Combination 2, SMD -0.05, 95%CI -0.24 to 0.14, *p* = 0.59) (Fig. 2). There was no significant heterogeneity between trials (I^2^ = 0%; p = 0.59–0.67). However, the effect estimates of one trial showed that men in the SDM group had significantly lower scores of decisional conflict on the factors contributing to uncertainty subscale (SMD -0.57, 95%CI -0.84 to − 0.29, *p* < 0.0001) (Table 4) [45].

## Discussion

In this systematic review with meta-analysis, the association of SDM with patient outcomes for decisions about PSA testing, as compared to usual care, is inconclusive. SDM is often discussed by policy makers and professional bodies as the best practice model for the physician-patient communication [46], and continues to be highly (guideline-) recommended for medical decisions about PC screening. Many HCP apply elements and features of SDM with a broader scope in their clinical practice, for example using patient-centred communication and patient activation strategies. We found, however, a very low volume of empirical research fulfilling the criteria for SDM in which various SDM interventions are compared to usual care (*n* = 4).

Although the interventions varied in characteristics and content, the study / interventions were aimed at and contained elements for fostering SDM. The outcomes of interest for this review also varied widely across studies in both quantity and reporting quality. While screening, knowledge and decisional conflict are the most reported SDM process-related outcomes, decisional regret, patient satisfaction or risk perception are scarcely reported, and no study reported on health outcomes. Moreover, no study fulfilled all the methodological quality criteria assessed. Besides sample sizes being modest, the studies are prone to high risk of bias mainly due to problems in the generation of the random sequence, blinding of outcome assessors, and having an attrition rate of at least 20% for some outcomes.

The evaluated literature represent in their majority White (76.6%), middle-aged (range: 54 to 63), married (76.6%) men from Western countries, mainly the USA, faced with decision-making about individualised PSA testing, who were recruited from general or internal medicine. Most men had PSA testing before the study and a great proportion reported a family history of PC. The participating HCP were family and internal medicine physicians in all studies and surprisingly there were no urologists at all. SDM interventions were mostly developed for and directed at men facing PSA screening decisions, included elements to inform and educate, and were delivered before the decision-making consultations. Finally, the results from this review are based on published data. This review should thus be considered within this context.

Despite this diversity and methodological limitations, our meta-analyses showed a small but significant effect of SDM interventions in improving knowledge of PC and screening, but no significant differences between SDM and usual care in reducing patient participation in PSA testing and decisional conflict. Interestingly, the patients from two RCTs were heavily (68–83%) screened before enrolment, and a larger than expected proportion of patients reported a family history of PC. This background information may be a factor influencing men’s decision for (less) PSA testing and the (lower) likelihood of men starting any decision-making process. Our meta-analyses showed the same direction of effect across studies however, and no significant between-study heterogeneity. Although 53.4% (*n* = 8) of the individual trial estimates showed no difference between SDM and usual care, 46.7% (*n* = 7) also suggest an association of SDM with improved outcomes. SDM may reduce or neutralise the tendency of physicians to order or recommend PSA testing and may improve the accuracy of patients’ perception of lifetime-risks for PC morbidity and mortality. In the latter [45], around 70% of men had an education of high school or less and men’s estimate of the lifetime risk of dying from PC was highly significant (RR 13.22, 95%CI 4.30 to 40.66; *p* < 0.00001). Although lower-literacy has been associated with an individual’s overestimation of risk perception [47], high-literacy individuals may also benefit from the educational interventions developed for low-literacy individuals [48]. Furthermore, an increased level of understanding and health knowledge are thought to positively influence decision-making and health behaviours. Remarkably, we found no evidence on the effects of SDM versus usual care on decisional regret and on very important health outcomes including mortality, quality of life, and symptoms. This absence of evidence does not mean that SDM does not have a beneficial effect on patient outcomes at individual or group levels however. Finally, men and physicians were the target of multifaceted interventions in only one RCT [41]. Of note, the included studies reported no cost data.

### Future research

Our findings are applicable to the development of future SDM interventions for decisions about PSA testing and our evaluation of the available evidence highlights a major knowledge gap. Future studies warrant further focus. Only a few studies fulfilled all SDM criteria suggesting that SDM is not yet fully adopted in practice despite guideline recommendations. Some guidance for SDM implementation may therefore be necessary. Whether patient activation without all the mandatory elements of SDM generates the same effects, and whether SDM is cost-effective compared to usual care remain questions for future research. Considering the levels of literacy and understanding may help explain the association between SDM and health outcomes. Future studies also need to address the barriers in the implementation of SDM e.g. physicians’ education, time consumption, patients’ responsibility and literacy. This could also guide SDM research in other areas of medical practice. Very importantly, the fact that at least 75% of the patients were Caucasian and married clearly indicates the need for assessing the impact of SDM among other racial and ethnic groups, and across different socio-economic strata. Future research could also help clarifying whether predefinition of study populations based on potential modifiers such as PSA pre-screening and family history of PC can impact the effect of SDM.

Current research could benefit from a more complete description of all interventions and outcome tools. Standardisation of the definition and objectives of SDM could lead to a clearer definition of the outcomes that are best for assessing SDM and its (clinical) effectiveness, and to guide the reasons for outcome selection. The outcomes used to measure the effectiveness of SDM is a topic under debate due to the individualisation of the SDM process. However, to understand the full impact of SDM, one needs to consider the type of decisions made and the extent to which SDM may actually affect health outcomes. Whether patients experience the health outcomes they expect, prefer or to which they feel unfavourably disposed could further guide the investigation of SDM. Future research should therefore address the absence of evidence on the effect of SDM on these outcomes. Future studies should also consider that interventions directed at both HCP and patients have been most effective in improving SDM-related and health outcomes [27]. Finally, larger and more methodologically sound studies could help confirming the findings from this review.

### Strengths and limitations

To our knowledge, this is the first systematic review about SDM compared to usual care for decisions about PC screening. Compared to other reviews [27], ours evaluates the effect of (higher quality) SDM implementation on both SDM-related and patient outcomes with a focus on PC testing. We searched for international literature with no restriction on countries or type of HCP. Our review also benefits from the inclusion of RCTs thus allowing the estimation of causal effects with lower risk of bias. In addition, our comprehensive search strategy comprised terms relevant to decision-making including SDM and DAs. We included studies published in English only and although the academic databases were searched up to March 2015, we made considerable efforts to identify all relevant studies. We also searched for grey literature by accessing the records retrieved from several sources between February and August 2016. By contacting authors between 2015 and 2017, we increased the chances of identifying more of the latest literature and full-texts with more complete data. Apart from using thorough electronic and manual searches, we conducted in-duplicate screening and study selection by applying a broad inclusion criteria at screening and full-text evaluation, and considered studies regardless of whether a specific decision was promoted.

We restricted our review to studies that fulfilled all SDM framework criteria [31] because of the continuing gaps in the conceptualisation and implementation of SDM. Our SDM framework is based on the SDM model by Charles et al. [29], the most prominent approach of viewing SDM with only one SDM concept [49] supporting the principle of bi-directional deliberation in keeping with Elwyn et al. [30]. This rigorous and focused approach allowed us to identify studies that integrated the key elements of SDM. The assessment was based on reported data, which cannot exclude the possibility of underreported SDM characteristics in other studies. Nevertheless, our review represents the results of studies with higher quality SDM implementation. Furthermore, to increase the precision of the effect estimates, we incorporated meta-analyses where possible, and assessed the risk of bias of individual studies.

The results from our systematic review are mainly limited by the quantity and methodological quality of the available literature. Only a few studies fulfilled the SDM framework and compared SDM to usual care. In addition, there is a dearth of data for nearly half of the outcomes of interest and the outcomes varied in the measurements used and reporting quality. The studies tend to focus primarily on SDM process-related outcomes and do not report on health outcomes. Thus, the few meta-analyses that we could incorporate contained two studies at most. Furthermore, the interventions’ content, especially usual care, were not fully described; and not only the studies were prone to high risk of bias but sample sizes were generally modest.

## Conclusions

A few studies that currently fulfil the criteria for SDM also assess the comparative effectiveness of SDM with usual care for decisions about PC screening. The studies comprise various SDM-fostering interventions and outcomes of variable reporting quality. SDM may improve knowledge and patient-perception of risk, and may reduce the tendency of physicians to recommend PSA testing. SDM may be similar to usual care in reducing patient participation in PSA testing, and in improving patient satisfaction and decisional conflict. There is insufficient evidence to support a clear association of SDM on patient-important and SDM-related outcomes for decisions about PSA testing. Future research needs to assess the clinical effectiveness of SDM using well-defined SDM interventions and outcomes. It should address the absence of evidence especially of health outcomes and costs.

## Additional files

Additional file 1: PRISMA checklist. (DOCX 43 kb)

Additional file 2: Search strategy for OVID Medline. (DOCX 18 kb)
