# Correction to: The effects of shared decision-making compared to usual care for prostate cancer screening decisions: a systematic review and meta-analysis

**DOI:** 10.1186/s12885-018-5029-7

**Published:** 2018-11-30

**Authors:** Nahara Anani Martínez-González, Stefan Neuner-Jehle, Andreas Plate, Thomas Rosemann, Oliver Senn

**Affiliations:** Institute of Primary Care, University of Zurich and University Hospital of Zurich, Pestalozzistrasse 24, CH-8091 Zurich, Switzerland

## Correction

Following publication of the original article [1], the authors notified us of a misleading data presentation in Table 4. The table’s sub header incorrectly presented the information in the first part of the table, BINARY DATA. We have therefore modified this sub header and added a second sub header to the table, corresponding to CONTINUOUS DATA.

The original publication has been corrected. Table 4 as initially published is presented below.

**Table 4:** Individual trial estimates not combined in meta-analyses

First author & publication year	Outcome	Measurement point	Intervention			Control			Effect estimate
			SDM	mean (SD)	Total (N)	Usual Care	mean (SD)	Total (N)	SMD (95 % CI)
BINARY DATA									
*Patient-reported ordering of screening*									
Krist, 2007 [43, 44] (Woolf, 2005)	patient-reported PSA tests ordered (patients’ exit questionnaires)	immediately after consultation	1) web-based DA	176	226	no pre-visit educational material and no DA during discussions with physicians	60	75	0.97 (0.85 to 1.11)
			2) paper version of DA in 1)	151	196		60	75	0.96 (0.84 to 1.10)
*Actual ordering of screening*									
Landrey, 2013 [42]	PSA tests order by clinicians (chart-documented)	following doctor’s appointment	flyer	85	136	no flyer	86	147	1.07 (0.88 to 1.29)
Krist, 2007 [43, 44] (Woolf, 2005)	physician-reported PSA tests ordered (chart-documented)	immediately after consultation	1) web-based DA	176	205	no pre-visit educational material and no DA during discussions with physicians	66	70	0.91 (0.84 to 0.99)
			2) paper version of DA in 1)	155	182		66	70	0.90 (0.83 to 0.98)
*Physicians’ recommendations: towards screening*									
Wilkes, 2013 [41]	doctor’s recommendations towards PSA screening: unannounced standardised patients (physicians’ questionnaires)	after clinic visit^b^	1) MD-Ed + A	16	36	CDC educational brochures on PC	34	43	0.56 (0.38 to 0.84)
			2) MD-Ed	24	41		34	43	0.74 (0.55 to 1.00)
*Physicians’ recommendations: neither nor against screening*									
Wilkes, 2013 [41]	doctors neither suggested nor recommended for or against PSA test: unannounced standardised patients (physicians’ questionnaires)	after clinic visit^b^	1) MD-Ed + A	18	36	CDC educational brochures on PC	6	43	3.58 (1.59 to 8.06)
			2) MD-Ed	14	41		6	43	2.45 (1.04 to 5.76)
*Patient-estimates of lifetime risks*									
Gatellari, 2003 [45]	how likely men were to give a correct estimate (within 2%) of the lifetime risk of dying from PC (correct answers over incorrect answers)	unclear (questionnaires mailed 3 days post-consultations)	32-page (3085-word) evidence-based booklet	55	104	968-word pamphlet by the Australian government	3	75	13.22 (4.30 to 40.66)
	how likely men were to give a correct estimate (within 10%) of the lifetime risk of developing PC (correct answers over incorrect answers)			59	104		18	108	3.40 (2.16 to 5.36)
CONTINUOUS DATA									
*Satisfaction with the visit*									
Wilkes, 2013 [41]	patient-reported satisfaction with the visit: planned visits (sum of 5 satisfaction items: 5 = least satisfied, 20 = most satisfied)	after clinic visit^b^	MD-Ed + A	18 (3.00)	102	CDC educational brochures on PC	18 (3.00)	291	0.00 (-0.23 to 0.23)
	patient-reported satisfaction with the visit: clinic visits by patients (sum of 5 satisfaction items: 5 = least satisfied, 20 = most satisfied)		MD-Ed	18 (2.00)	188		18 (3.00)	291	0.00 (-0.18 to 0.18)
*Men’s views towards screening*									
Gatellari, 2003 [45]	men’s views weighted towards or against reasons for having PSA testing (Scoring -5 to 5. Positive: weighting for; Higher: stronger weighting for; Negative: weighting against; Lower: stronger weighting against)^b^	unclear (questionnaires mailed 3 days post-consultations)	32-page (3085-word) evidence-based booklet	1.70 (1.58)	106	968-word pamphlet by the Australian government	1.4 (1.59)	108	0.19 (-0.08 to 0.46)
*Decisional conflict*									
Gatellari, 2003 [45]	decisional conflict (9-item factors contributing to uncertainty scale; higher scores = greater decisional conflict)	unclear (questionnaires mailed 3 days post-consultations)	32-page (3085-word) evidence-based booklet	21.60 (4.73)	106	968-word pamphlet by the Australian government	24.3 (4.77)	108	-0.57 (-0.84 to -0.29)

*PC* Prostate Cancer, *SDM* Shared Decision-Making, *MD-Ed + A* Physician Education and patient Activation, *MD-Ed* Physician Education, *DA* Decision Aid, *CDC* Centers for Disease Control and Prevention, *PSA* Prostate Specific Antigen, *n* number of patients with events or number of events, *N* total number of patients per group, *RR* Relative Risk, *SD* Standard Deviation, *SMD* Standard Mean Difference, *CI* Confidence Intervals

^a^Questionnaire adapted from an attitudinal measure of the mammography screening instrument

^b^Men followed-up in 6-16 weeks depending on the timing of the standardised visit: about 6 weeks after the intake survey for control physicians, 6-10 weeks for MD-Ed physicians, and 6-16 weeks for MD-Ed+A physicians
