# Supplementary material for: The effects of shared decision-making compared to usual care for prostate cancer screening decisions: a systematic review and meta-analysis
Source: BMC Cancer. 2018 Oct 22;18:1015. doi: 10.1186/s12885-018-4794-7 (PMC6196568; doi:10.1186/s12885-018-4794-7)
Supplement: Supplementary file 2 — Search strategy for OVID Medline. (DOCX 18 kb) [file 12885_2018_4794_MOESM2_ESM.docx]

**Additional file 2**. Search strategy for OVID Medline.

|  |
| --- |

| **Item** | **Searches** |
| --- | --- |
| 1 | exp Decision Making/ or Decision Making, Organizational/ or Decision Trees/ or Decision Making/ or Decision Support Techniques/ or Decision Support Systems, Clinical/ or Decision Making, Computer-Assisted/ or exp Computer-Assisted Instruction/ or exp Patient Participation/ or exp Professional-Patient Relations/ or exp "Attitude of Health Personnel"/ or Counseling/ or exp Health Communication/ |
| 2 | exp Informed Consent/ |
| 3 | (choice behavior or decision making or shared decision making).mp,tw. |
| 4 | (informed adj3 (consent or choice* or decision*)).mp,tw. |
| 5 | ((decision* or decid*) adj4 (support* or aid* or tool* or instrument* or technolog* or technique* or system* or program* or algorithm* or process* or method* or intervention* or material*)).mp,tw. |
| 6 | (decision adj3 (board* or guide* or counseling)).mp,tw. |
| 7 | (computer* adj4 decision making).mp. |
| 8 | (patient adj3 (participation or involvement or cent#d care)).mp,tw. |
| 9 | ((risk communication or risk assessment or risk information) adj4 (tool* or method*)).mp,tw. |
| 10 | interact* health communication*.mp,tw. |
| 11 | (interact* adj (internet or online or graphic* or booklet*)).mp,tw. |
| 12 | (interact* adj4 tool*).mp,tw. |
| 13 | ((interact* or evidence based) adj3 (risk information or risk communication or risk presentation or risk graphic*)).mp,tw. |
| 14 | adaptive conjoint analys#s.mp,tw. |
| 15 | or/1-14 |
| 16 | (Prostat* adj3 (Neoplasm* or Cancer or tumo?r* or carcinoma)).mp,tw. |
| 17 | exp Prostatic Neoplasms/ |
| 18 | 16 or 17 |
| 19 | 15 and 18 |
| 20 | (letter or letter$ or editorial or historical article or anecdote or commentary or note or case report$ or case study).pt,sh. |
| 21 | (animals not humans).sh. |
| 22 | 20 or 21 |
| 23 | 19 not 22 |
| 24 | exp Randomized Controlled Trial/ or exp clinical trial/ |
| 25 | randomized controlled trial.pt. |
| 26 | randomized controlled trial.sh. |
| 27 | controlled clinical trial.pt. |
| 28 | random allocation.sh. |
| 29 | double blind method.sh. |
| 30 | single blind method.sh. |
| 31 | or/24-30 |
| 32 | 31 not 22 |
| 33 | exp clinical trial/ or exp Clinical Trials as Topic/ |
| 34 | clinical trial.pt. |
| 35 | ((singl$ or doubl$ or trebl$ or trpl$) adj25 (blind$ or mask$)).ti,ab. |
| 36 | (clin$ adj25 trial$).ti,ab. |
| 37 | (random$ or placebo$).ti,ab. |
| 38 | (PLACEBO or RESEARCH DESIGN).sh. |
| 39 | or/33-38 |
| 40 | 39 not 22 |
| 41 | 40 not 32 |
| 42 | exp EVALUATION STUDIES/ |
| 43 | (comparative study or follow up studies or prospective studies).sh. |
| 44 | (control$ or prospectiv$ or volunteer$).ti,ab. |
| 45 | or/42-44 |
| 46 | 45 not 22 |
| 47 | 46 not (32 or 41) |
| 48 | 23 and (32 or 41 or 47) |
